# Real-Time Tracking of *Ex Vivo*-Expanded Natural Killer Cells Toward Human Triple-Negative Breast Cancers

**DOI:** 10.3389/fimmu.2018.00825

**Published:** 2018-05-02

**Authors:** Tung Nguyen Thanh Uong, Kyung-Hwa Lee, Sung-Ja Ahn, Kyung Won Kim, Jung-Joon Min, Hoon Hyun, Mee Sun Yoon

**Affiliations:** ^1^Department of Radiation Oncology, Chonnam National University Hwasun Hospital, Chonnam National University Medical School, Gwangju, South Korea; ^2^Department of Biomedical Science, Chonnam National University Graduate School, Gwangju, South Korea; ^3^Department of Pathology, Chonnam National University Hwasun Hospital, Chonnam National University Medical School, Gwangju, South Korea; ^4^Department of Nuclear Medicine, Chonnam National University Hwasun Hospital, Hwasun, South Korea; ^5^Department of Biomedical Sciences, Chonnam National University Medical School, Gwangju, South Korea; ^6^Research Center for Cancer Immunotherapy, Chonnam National University Hwasun Hospital, Jeollanam-do, South Korea

**Keywords:** natural killer cells, ESNF13, near-infrared fluorophores, MDA-MB-231 tumor-bearing mouse, optical imaging, *in vivo* tracking

## Abstract

**Introduction:**

*Ex vivo*-expanded natural killer (NK) cells are a potential candidate for cancer immunotherapy based on high cytotoxicity against malignant tumor cells. However, a limited understanding of the migration of activated NK cells toward solid tumors is a critical dilemma in the development of effective and adoptive NK cell-based immunotherapy.

**Methods:**

*Ex vivo*-expanded NK cells from healthy donors were stained with near-infrared fluorophores at different concentrations. NK cell proliferation and cytotoxicity were assessed using a WST-8 assay, while the expression levels of surface molecules were analyzed by flow cytometry. To investigate the biodistribution of NK cells in both normal and tumor-bearing NSG mice, NK cells labeled with ESNF13 were subjected to NIR fluorescence imaging using the Mini-FLARE imaging system. Finally, mice were sacrificed and histopathological tests were performed in resected organs.

**Results:**

The signal intensity of ESNF-stained NK cells was long-lasting at 72 h using concentrations as low as 0.04 µM. At a low dose range, ESNF13 did not affect NK cell purity, expression levels of surface receptors, or cytotoxic functions against MDA-MB-231 cancer cells. *Ex vivo*-expanded NK cells labeled with ESNF13 had a 4-h biodistribution in non-tumor-bearing NSG mice that mainly localized to the lungs immediately after injection and then fully migrated to the kidney after 4 h. In an MDA-MB-231 tumor-bearing NSG mice with extensive metastasis in both lungs, the fluorescence signal was dominant in both lungs and steady at 1, 2, and 4 h post-injection. In a early phase of tumor progression, administered NK cell migrated to the lungs and tumor sites within 30 min post-injection, the signal dominated the tumor site after 1 h, and remained steady at 4 h.

**Conclusion:**

Optical imaging with NIR fluorophore ESNF13 is a highly sensitive, applicable, and inexpensive method for the real-time tracking of *ex vivo*-expanded NK cells both *in vitro* and *in vivo*. Administered NK cells had different patterns of NK cell distribution and accumulation to the tumor site according to tumor progression in triple-negative breast cancer xenograft models.

## Introduction

Cancer immunotherapy using *ex vivo*-expanded natural killer (NK) cells is promising for the treatment of hematologic cancer and solid tumors (1–3). Although activated and expanded NK cells are highly cytotoxic to malignant tumor cells, little is known about the NK cells’ distribution, persistence, and migration to tumor sites *in vivo*. Understanding the migration pattern of immune cells, especially in solid tumors, could prove critical to the development of effective immunotherapy (4).

Optical imaging with near-infrared (NIR) fluorophores is easily applicable, fast, inexpensive, and provides highly sensitive non-invasive imaging in preclinical and clinical settings (5–7), representing a useful imaging modality for *in vivo* tracking of NK cells. One preclinical study has evaluated human NK-92 cell lines labeled with NIR dye in a human prostate cancer xenograft (7), but there is little study that targets *ex vivo*-expanded NK cells from primary cells extracted from peripheral blood mononuclear cells (PBMCs). Adoptively administered NK cells have been labeled with radioactive markers (^11^C or^18^ F) for positron emission tomography (PET) (8, 9) and with ^111^In-oxine for single photon emission computerized tomography (SPECT) in solid human tumors (10). Optical imaging, compared with these molecular imaging techniques such as PET and SPECT, is benefited by real-time *in vivo* tracking of NK cells immediately after intravenous NK injection without radiation exposure. Repeated radiation exposure, decay of the labeled radioactive dye, and starvation for imaging work-up can be toxic to living animals, resulting in limited preclinical use. Therefore, non-invasive NIR fluorescence imaging using cell tracking agents like ESNF13 has been used to monitor the location of inoculated cancer cells *in vivo*. The lipophilic NIR cyanine fluorophore ESNF13 was previously developed for the longitudinal monitoring of cell proliferation and differentiation with low cytotoxicity, high optical properties, and low background outside cells. Unlike other tracking techniques, ESNF13 requires facile and simple procedures for intracellular trafficking (11, 12).

In triple-negative breast cancer (TNBC), high tumor infiltrating lymphocytes and programmed cell death ligand 1 receptor (PD-L1) on tumor cells or in the tumor microenvironment predict the response to chemotherapy (13) and the clinical activity of anti-PD-1 immune check-point inhibitors (14, 15), thereby suggesting a potential candidate for the clinical development of highly cytotoxic NK cell-based immunotherapy. The effective migration of *ex vivo*-expanded NK cells in targeting cancer cells may enhance the immune response to TNBC, resulting in improved outcomes. Accumulation of NK cells can be easily determined by optical imaging in preclinical settings. Furthermore, optical imaging can potentially be used to guide the design of candidates, tumor types, various genetically modified NK cell types, and optimal treatment modality combination schedules.

In this study, we investigated a novel approach for the real-time tracking of *ex vivo-*expanded NK cells that are labeled with NIR fluorophores. To the best of our knowledge, this is the first study to identify NIR fluorophores for labeling *ex vivo*-expanded NK cells using optical imaging. We investigated whether NIR fluorophores influence the proliferation and cytotoxicity of *ex vivo* NK cells and to determine the biodistribution and accumulation at the tumor site of NK cell-injected NOD-SCID-IL2 receptor γ^null^ (NSG) mice bearing human TNBC.

## Materials and Methods

### Reagents and Antibodies

The anti-human monoclonal antibodies (mAbs) for flow cytometry were fluorescein iso-thiocyanate (FITC)-conjugated CD3, phycoerythrin-cyanine 5-conjugated CD56, phycoerythrin (PE)-conjugated CD11a, PE-conjugated CD16, PE-conjugated CD107a, PE-conjugated CD279, PE-conjugated CD335, PE-conjugated CD337, PE-conjugated CD314 and IgG1 isotype control, purchased from BD Biosciences (San Jose, CA, USA), and anti-human Abs PE-conjugated CD159c, PE-conjugated CD159a, PE-conjugated IgG1 and PE-conjugated IG2A, purchased from R&D systems (Minneapolis, MN, USA). The following recombinant human interleukins, rhIL-2, rhIL-15, and rhIL-21 (PeproTech, Rocky Hill, NJ, USA), were used to expand the NK cells. Vita-Orange Cell Viability Reagent (WST-8; Biotool, Houston, TX, USA) was used for the cytotoxicity assay. Matrigel (BD Biosciences, San Jose, CA, USA), the reconstituted basement membrane matrix, was used for inducing MDA-MB-231 tumor growth in NSG mice. The use of animals for this study was approved by the Institutional Animal Care and Use Committee of Chonnam National University.

### Cell Lines

The human breast cancer cell line MDA-MB-231 was obtained from the American Type Culture Collection (Manassas, VA, USA). The MDA-MB-231 cells were cultured in RPMI1640 media supplemented with 10% inactivated fetal bovine serum (FBS), 100 U/mL penicillin, and 100 µg/mL streptomycin (all from Invitrogen, Carlsbad, CA, USA). Conventional K562 cells, which were used as feeder cells for the NK cell culture, were cultured in RPMI1640 medium containing 10% FBS, 100 U/mL penicillin, 100 µg/mL streptomycin, and 4 mmol/L l-glutamine. All of the cell lines were incubated at 37°C in a humidified 5% CO_2_ incubator.

### Mouse and MDA-MB-231 Xenograft Model

Six- to nine-week-old immunodeficient NOD.Cg-*Prkdc^scid^IL2rg^tm1Wjl^/*SzJ (NSG) mice (Jackson Laboratory, Bar Harbor, ME, USA) were used in this study. The mice were maintained under sterile conditions in an animal lab at the Chonnam National University Hwasun Hospital (South Korea). All animals were fed with free alfalfa feed 72 h before optical imaging. The human breast cancer MDA-MB-231 xenograft models were made to investigate NK cell trafficking *in vivo*. The MDA-MB-231 cancer cells were harvested by centrifugation at 1,300 rpm and washed two times with phosphate buffered saline (PBS). The MDA-MB-231 cancer cells (1 × 10^7^ cells) were then mixed with Matrigel on ice at a ratio of 1:1. The mixture was then subcutaneously injected into the right-back leg of the NSG mice. All MDA-MB-231 tumor-bearing mice were maintained for at least 30 days before use in further experiments. The use of animals for this study was approved by the Institutional Animal Care and Use Committee of Chonnam National University.

### NK Cell Expansion

Peripheral blood mononuclear cells from different healthy donors were isolated using Lymphoprep solution (Axis-Shield) and centrifugation at 2,300 rpm for 25 min as described (16). PBMCs were co-cultured with 100 Gy gamma ray-irradiated conventional K562 cells in a 24-well plates with RPMI1640 medium (10% FBS, 100 U/mL penicillin, 100 µg/mL streptomycin, and 4 mmol/L l-glutamine) containing 5 ng/mL rhIL-21 and 10 U/mL rhIL-2. The medium was exchanged on day 3 and day 5 with fresh medium containing 10 U/mL rhIL-2. From day 7, the medium was exchanged every 2 days with new medium in the presence of 100 U/mL rhIL-2 and 5 ng/mL rhIL-15. Expanded NK cells were harvested on day 14 and used for further experiments.

### Staining NK Cells With ESNF13 NIR Fluorophores

ESNF13 was synthesized as described previously (11) and dissolved in dimethyl sulfoxide (DMSO) to generate 10 mM stock solutions. Expanded NK cells were harvested day 14 and washed with 1× PBS. NK cells were then stained with different concentrations of ESNF13 dye (1, 0.4, 0.2, 0.1, and 0.04 µM) in the dark and incubated for 10 min at 37°C in a humidified 5% CO_2_ incubator. After incubation, the NK cells were centrifuged at 1,300 rpm for 3 min at room temperature. The supernatant was discarded and NK cells were washed three times with 1× PBS until the supernatant became clear. Stained NK cells were then used for further analysis.

### NK Cell Proliferation by Cell Counting and by WST-8 Assay

Natural killer cells (2 × 10^6^ cells) stained with 0.1 and 0.2 µM ESNF13 dye were cultured in 24-well plates with RPMI1640 medium (10% FBS, 100 U/mL penicillin, 100 µg/mL streptomycin, and 4 mmol/L l-glutamine) in the presence of 100 U/mL rhIL-2 and 5 ng/mL rhIL-15. At different time points (0, 24, 48, and 72 h), the stained NK cells were harvested by centrifugation at 1,300 rpm for 3 min at room temperature. The cells were then counted using Trypan Blue stain (Gibco), a hemocytometer (Marienfeld), and a microscope (Leica). The effect of the ESNF13 NIR fluorophores on NK cell proliferation was analyzed using a WST-8 assay. Expanded NK cells (2 × 10^5^ cells) were stained with ESNF13 dye (1, 0.4, 0.2, 0.1, and 0.04 µM) and cultured with RPMI1640 medium without phenol red (10% FBS, 100 U/mL penicillin, 100 µg/mL streptomycin, and 4 mmol/L l-glutamine) in the presence of 100 U/mL rhIL-2 and 5 ng/mL rhIL-15 on irradiated flat type 96-well plates. After different time points (0, 24, 48, and 72 h), a control group (2 × 10^5^ freshly cultured NK cells) were added and then 10 µL WST-8 was loaded into each well, and the plate was incubated for 1 h. After incubation, the plate was placed on ice for 2 min to stop the WST-8 reaction, followed by centrifugation 1,300 rpm for 3 min at 4°C. The supernatants were transferred to a new plate, and the percentage of lysed NK cells was analyzed at 450 nm using an Infinite M200 Pro 96-well plate reader.

### Flow Cytometry

The effect of ESNF13 dye on the expression of adhesion molecules (LFA-1), antibody-dependent cell-mediated cytotoxicity (CD16), programmed cell death (PD-1), degranulation marker (CD107a), and activatory (NKp30, NKp46, NKG2D, and NKG2C) and inhibitory (NKG2A) receptors on the surface of expanded NK cells was analyzed by flow cytometry. Expanded NK cells (2 × 10^6^ cells) were stained with 0.1 and 0.2 µM ESNF13 and incubated for various lengths of time (0, 24, 48, and 72 h). NK cells labeled with dye were harvested and stained with mAbs specific for different types of surface receptors. After being washed with PBS and 5% bovine serum albumin (BSA), all cells were stained on ice for 15 min. The stained cells were washed again with PBS and 5% BSA and then fixed with 2% paraformaldehyde. The stained and fixed cells were analyzed using a FACSCalibur (BD Biosciences, San Jose, CA, USA). Data were collected using BD CellQuest Pro software (BD Biosciences, San Jose, CA, USA).

### Optical Imaging of NK Cells *In Vitro*

Expanded NK cells on day 14 (2 × 10^5^ cells) were stained with ESNF13 dye at different concentration (2, 1, 0.4, 0.2, 0.1, and 0.04 µM), and were cultured on irradiated flat type 96-well plates for different time points (0, 24, 48, and 72 h). After each time points, the NIR imaging for stained NK cells was performed on a four filter set Nikon Eclipse Ti-U inverted microscope system. The microscope was equipped with a 100 W halogen lamp, NIR-compatible optics, and a NIR-compatible 10× Plan Fluor objective lens (Nikon, Seoul, South Korea). Image acquisition and analysis were performed using NIS-Elements Basic Research software (Nikon, Seoul, South Korea). The NIR filter set composed of 650 ± 22 nm excitation filter, 675 nm dichroic mirror, and 710 ± 25 nm emission filter was used to detect ESNF13 signals in the NK cells. All NIR fluorescence images had identical exposure times and normalization.

### Cytotoxicity Assay

The NK cells stained with ESNF13 (0.1 and 0.2 µM) were used in cytotoxicity assays to investigate the effect of the ESNF13 dye on the function of expanded NK cells. The 4-h cytotoxicity of the stained NK cells toward the MDA-MB-231 cancer cell line was investigated using a WST-8 proliferation assay at each time point after staining with ESNF13 (0, 24, 48, and 72 h). The cytotoxicity was investigated at various time intervals (0, 24, 48, and 72 h) after the NK cells were stained with different concentrations of ESNF13 dye (0.2 and 0.1 µM). The NK cells were harvested and then co-cultured with MDA-MB-231 cancer cells (5 × 10^4^) at an E:T (effector to target cell ratio) of 0.5:1 in irradiated flat type 96-well plates for 3 h. Subsequently, 10 µL WST-8 was added to each well (except for control wells containing only media) and the plate was incubated for 1 h. After incubation, the percentage of cancer cells lysed by NK cells was analyzed as previously described (17).

### Interferon-γ (IFN-γ) ELISA

The level of soluble IFN-γ was estimated using the BD OptEIA™ Human IFN-γ ELISA Set (BD Science, San Jose, CA, USA). NK cells stained with different concentration of ESNF13 NIR dye (0.1 and 0.2 µM) were harvested at various time intervals (0, 24, 48, and 72 h) and co-cultured with MDA-MB-231 cancer cells (5 × 10^4^ cells; E:T ratio, 0.5:1) on irradiated flat type 96-well plates for 24 h at 37°C in a humidified 5% CO_2_ incubator. After incubation, the supernatants were isolated by centrifugation and used to estimate the level of IFN-γ released by NK cells (ELISA was performed as per the manufacturer’s instructions).

### NIR Fluorescence Imaging System

*In vivo* NIR fluorescence imaging was performed using the Mini-FLARE imaging system as described previously (12). Briefly, the system consists of two wavelength-separated light sources: a “white” LED light source, generating 26,600 lux of 400–650 nm light to illuminate the surgical field and a NIR LED light source, generating 1.08 mW/cm^2^ of 656–678 nm fluorescence excitation light. White light and NIR fluorescence images were acquired simultaneously and displayed in real-time using custom-designed optics and software.

### Biodistribution of NK Cells on Non-Tumor-Bearing NSG Mice

To determine the biodistribution of NK cells *in vivo*, the expanded NK cells (2 × 10^7^ cells) from day 14 to 17 were stained with 0.2 µM ESNF13 NIR fluorophores and 6- to 9-week-old NSG mice were intravenously injected with stained NK cells. The mice were analyzed using a NIR optical imaging system in a time-dependent manner. Mice were sacrificed every 30 min, 1 h, and 4 h after injection and the optical imaging signal was taken for each organ. Then, resected organs were fixed in 10% neutral-buffered formalin for 3 days. Then, the organs were dissected, embedded in paraffin, and stained with hematoxylin for histopathological evaluation. Immunohistochemistry using anti-human CD56 antibody (dilution 1:50, code M7304, DakoCytomation, Glostrup, Denmark) and anti-HLA class 1 antibody (dilution 1:800, code ab70328, Abcam, Cambridge, UK) was performed on the same tissue blocks. As CD56 was used for confirmation the present of human NK cells in the resected organs, while HLA class 1 was used for the confirmation of human cancer cells MDA-MB-231 in primary tumor and metastasis organs. 3 µm-thick tissue sections were submitted for staining using an automated immunostainer (Bond-maX DC2002, Leica Biosystems, Bannockburn, IL, USA).

### Tumor and Lung-Meta Imaging in MDA-MB-231 Tumor-Bearing Mice

The MDA-MB-231 tumor-bearing mice (31–44 days after tumor injection) were used to investigate the trafficking of expanded NK cells in tumor models. The NK cells stained with 0.2 µM ESNF13 dye (2 × 10^7^ cells) were intravenously injected into MDA-MB-231 tumor-bearing mice. All mice were analyzed using a NIR optical imaging system as mentioned above. Subsequently, mice were dissected for study and each organ and tumor were analyzed using the optical imaging signal. All specimens were sent to a pathology lab for further experiments.

### Statistical Analysis

Statistical data were analyzed using a paired sample *t*-test and analysis of variance (ANOVA) with *p*-values <0.05 considered significant. *Post hoc* analysis using a Tukey’s test was performed to confirm the differences between groups revealed by ANOVA. The expression of NK cell receptors was analyzed using WinMDI. All statistical analyses were performed using SPSS (SPSS Inc., Chicago, IL, USA).

## Results

### Optical Imaging of NK Cells *In Vitro*

**Figure 1:**
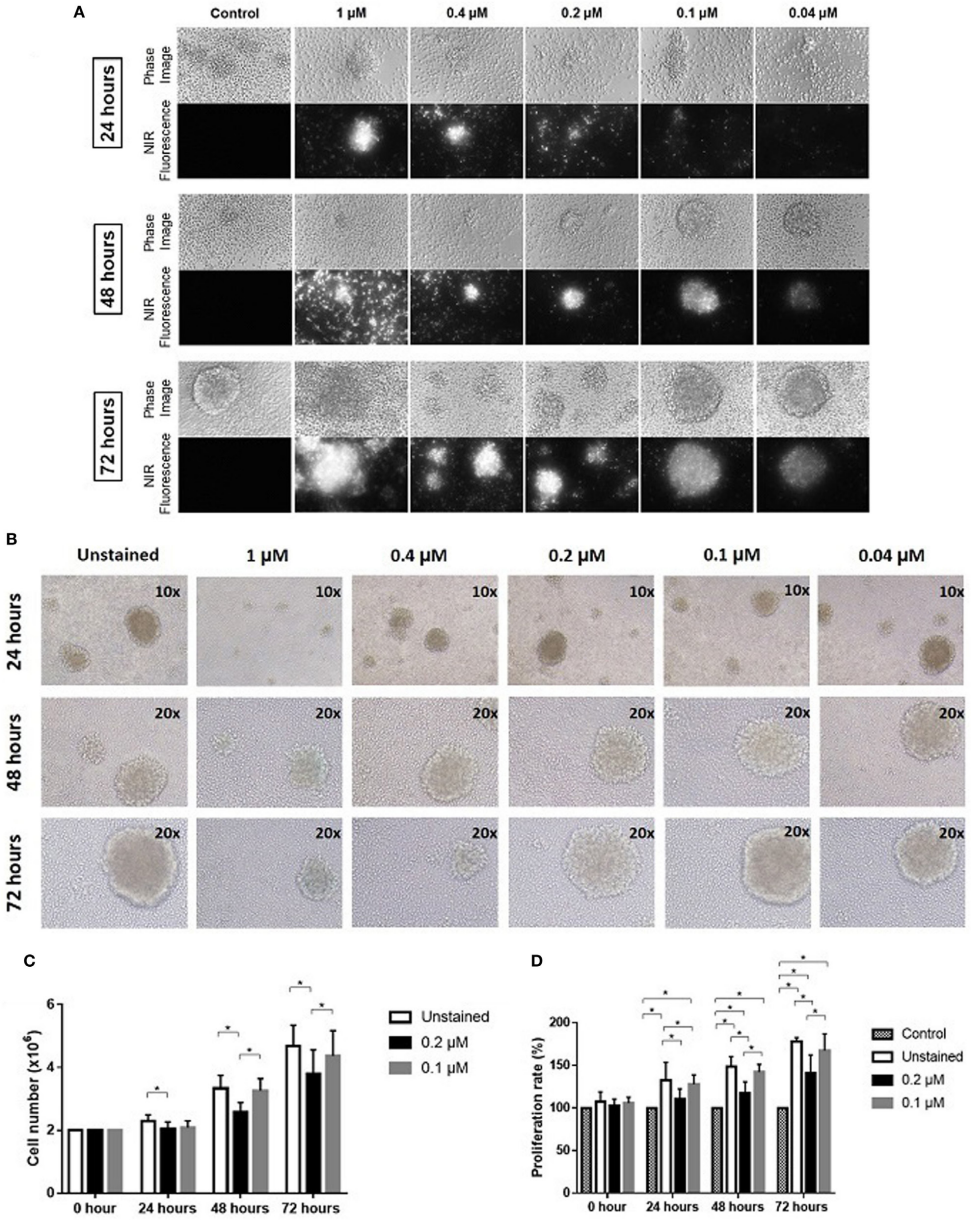
Optical imaging of near-infrared (NIR) fluorophore-stained natural killer (NK) cells *in vitro*. Expanded NK cells were stained with ESNF13 at different concentrations and evaluated at each designated time point. **(A)** Optical imaging of NK cells stained with NIR fluorophore ESNF13 at different concentrations was performed using a Nikon Eclipse T*i*-U inverted microscope system. **(B)** ESNF13-stained NK cells were pictured using an Olympus IX71 microscope. **(C)** Proliferation of ESNF13-stained NK cells by cell counting. The bar diagrams represent the number of NK cells (million cells). **(D)** Proliferation of NK cells as determined by a WST-8 proliferation assay. The bar diagrams present the proliferation percentage (%) compared with the control group. Data represent the mean ± SD from three independent experiments. Asterisk (*) indicates *p* < 0.05 in paired sample *t*-test analysis.

Expanded NK cells were stained with ESNF13 dye at different concentrations (1, 0.4, 0.2, 0.1, and 0.04 µM) and pictured using a Nikon Eclipse T*i-*U inverted microscope system. The fluorescent signal was still visible under immunofluorescent microscopy at concentrations as low as 0.04 µM. In addition, the signal intensity lasted at least 72 h at all of the assessed concentrations (Figure 1A).

### The Effect of NIR Fluorophores on NK Cell Proliferation *In Vitro*

Expanded NK cells from day 14 to 17 with purity over 90% were stained with ESNF13 NIR fluorophores at different concentrations to investigate the effect of the dye on NK cell proliferation. ESNF13 dye, at concentrations higher than 0.4 µM, inhibited NK cell cluster formation (Figure 1B). ESNF13 dye at a concentration of 0.1 µM did not affect the proliferation of expanded NK cells, demonstrated by both cell counting and the WST-8 proliferation assay (Figures 1B–D). Meanwhile, the NK cells stained with 0.2 µM ESNF13 dye showed slower proliferation than the unstained NK cells at 24 h (*p* = 0.036), 48 h (*p* = 0.027), and 72 h (*p* = 0.02), as determined by a cell count test (Figure 1C). In the WST-8 assay, 0.2 µM of ESNF13 dye delayed cell proliferation at 24 h (*p* < 0.05), 48 h (*p* = 0.01), and 72 h (p = 0.01) (Figure 1D).

### Purity and Surface Receptor Expression in NIR Fluorophores-Stained NK Cells

**Figure 2:**
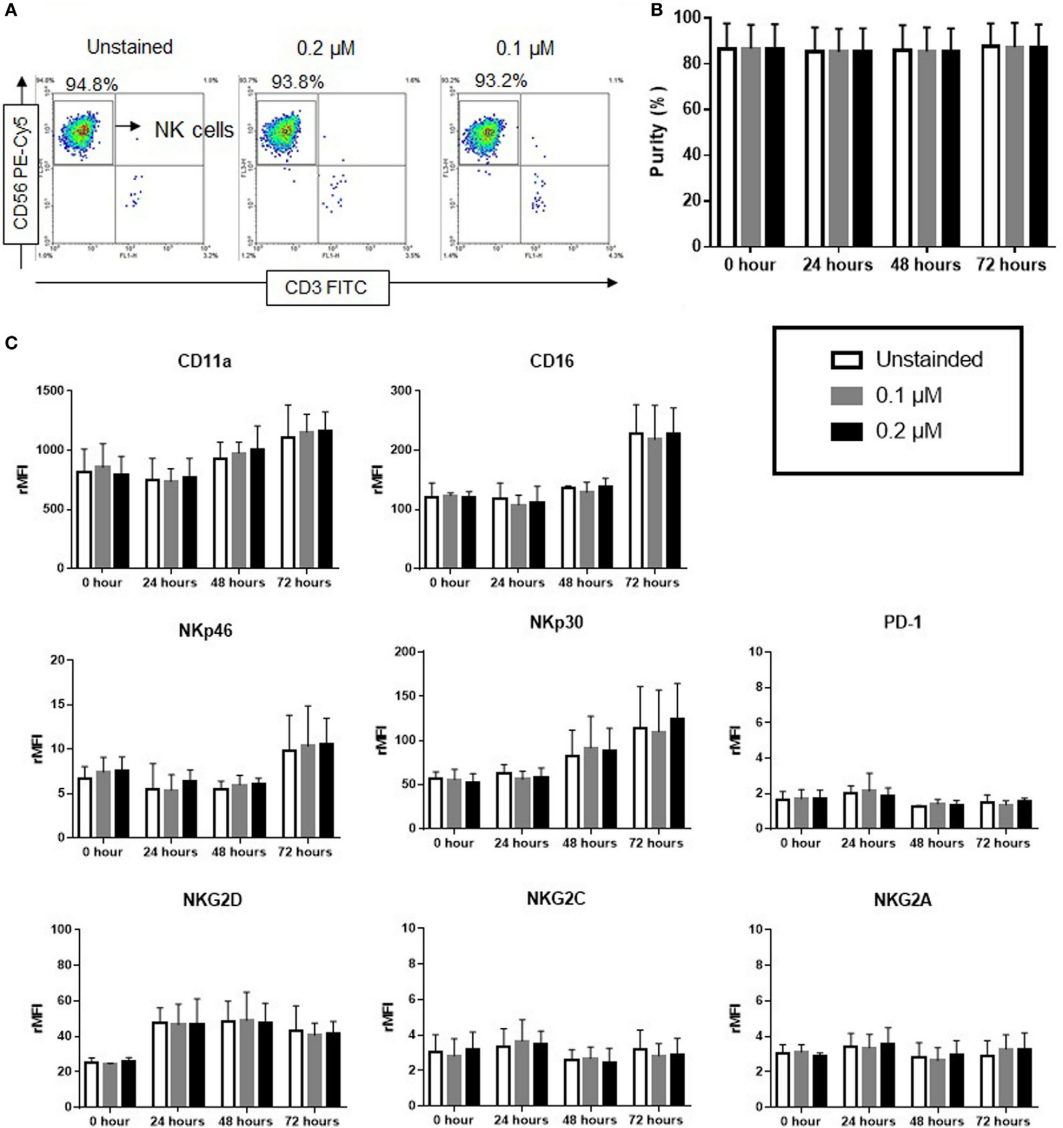
Effect of ESNF13 on natural killer (NK) cells purity and expression of surface receptors. Expanded NK cells were stained with ESNF13 at different concentrations and the resultant impact on purity and the expression of surface receptors were investigated using flow cytometry. **(A)** The representative dot plot examples of stained and unstained CD56^+^CD3^−^ NK cells after 72 h. **(B)** Purity of NK cells examined in a time-dependent manner. The bar diagrams represent the averaged purity percentage obtained from three independent experiments. **(C)** Surface receptor expression of ESNF13-stained NK cells. The bar diagrams represent the ratio of the mean fluorescent intensity values of surface receptors expressed as the means ± SD from three independent experiments.

The effect of ESNF13 NIR fluorophores on NK cell purity and phenotype was evaluated in a time-dependent manner using flow cytometry. 0.1 and 0.2 µM of ESNF13 were used for our experiments because higher concentrations inhibited NK cells proliferation (Figure 1). ESNF13 dye did not affect the purity of NK cells at concentrations of 0.1 and 0.2 µM until 72 h (Figures 2A,B and Figure S1 in Supplementary Material). The expression of PD-1 receptors did not change after cell staining with the dye. In addition, the expression of other receptors, including activating receptors (CD16, NKp30, NKp46, NKG2D, and NKG2C), inhibitory receptors (NKG2A), and adhesion receptors (LFA-1), was not significantly different after cell staining with 0.1 and 0.2 µM ESNF13 (Figure 2C).

### Fluorescent Signal Intensity Between Living and Dead NK Cells Stained With NIR Fluorophores

**Figure 3:**
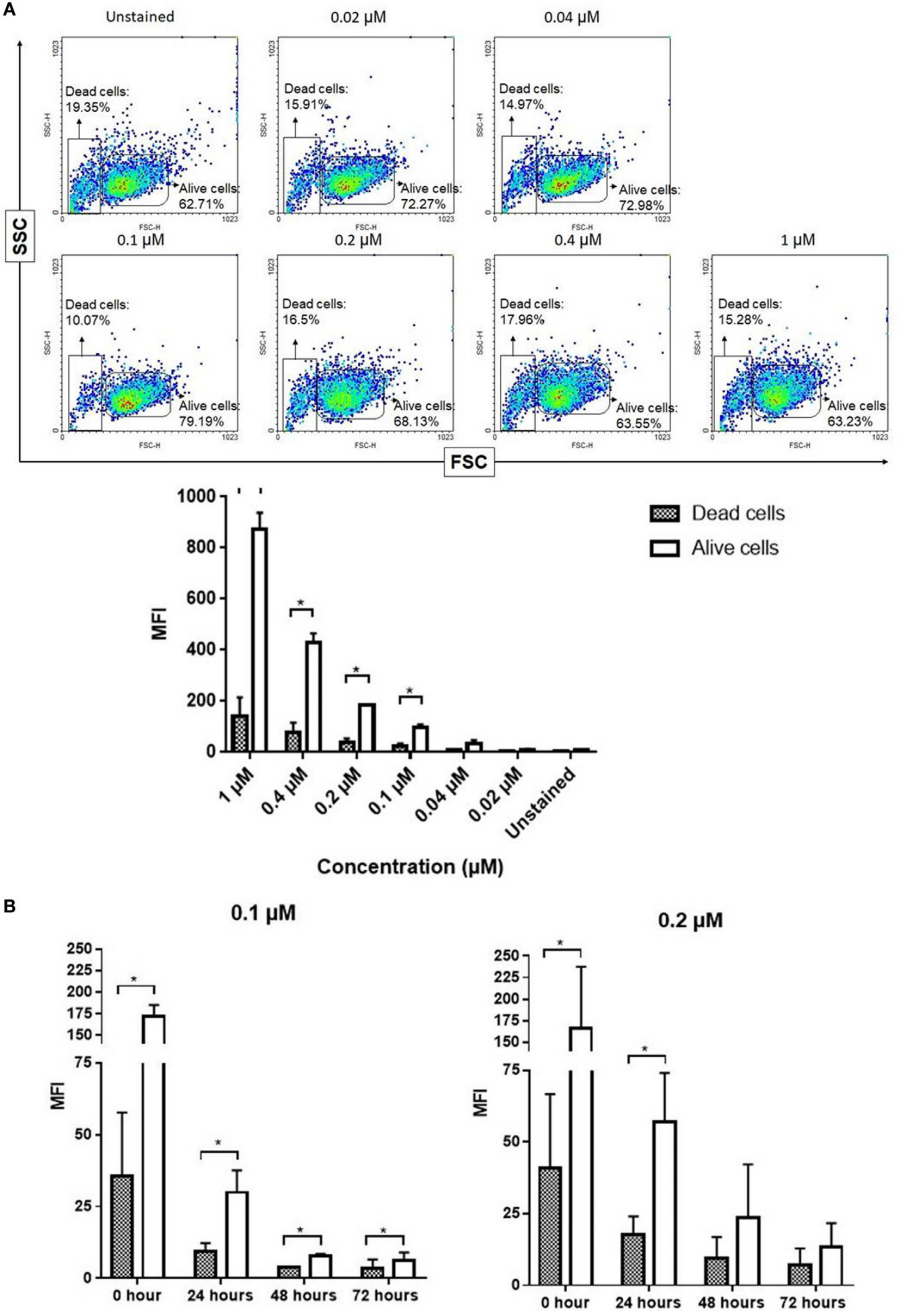
Fluorescent signal intensity between alive and dead natural killer (NK) cells stained ESNF13. **(A)** Expanded NK cells were stained with different concentrations of ESNF13 and the dye intensity of alive and dead cell populations were analyzed by flow cytometry immediately after staining. Representative flow cytometry density plots (top) and the mean fluorescent intensity values (bottom). **(B)** Expanded NK cells were stained with ESNF13 and the dye intensity of alive and dead cell populations assessed using flow cytometry was compared in a time-dependent manner. The bar diagrams represent the mean fluorescent intensity values. Data represent the mean ± SD from three independent experiments. Asterisk (*) indicates *p* < 0.05 in paired sample *t*-test analysis.

To evaluate the fluorescence intensity by flow cytometry, living and dead NK cells stained with NIR fluorophore ESNF13 were gated and analyzed (Figure S2 in Supplementary Material). Expanded NK cells stained with ESNF13 at different concentrations were analyzed by flow cytometry immediately after staining (Figure 3A). The fluorescence intensity of living ESNF13-stained NK cells was significantly increased in comparison with dead cells at concentrations of 1, 0.4, 0.2, and 0.1 µM. To determine whether the fluorescence intensity changed in a time-dependent manner, the fluorescence intensity was assessed using 0.1 and 0.2 µM ESNF13. At 0.2 µM ESNF13, the living cells had a significantly higher intensity at 0 h (*p* = 0.047), and 24 h (*p* = 0.024). Living cells stained with 0.1 µM ESNF13 had significantly higher MFI compared with the unstained cells at 0 h (*p* = 0.023), 24 h (*p* = 0.026), 48 h (*p* = 0.027), and 72 h (*p* = 0.037) (Figure 3B).

### NIR Fluorophores-Stained NK Cell Cytotoxicity and IFN-γ Production in Human Breast Cancer Cells *In Vitro*

**Figure 4:**
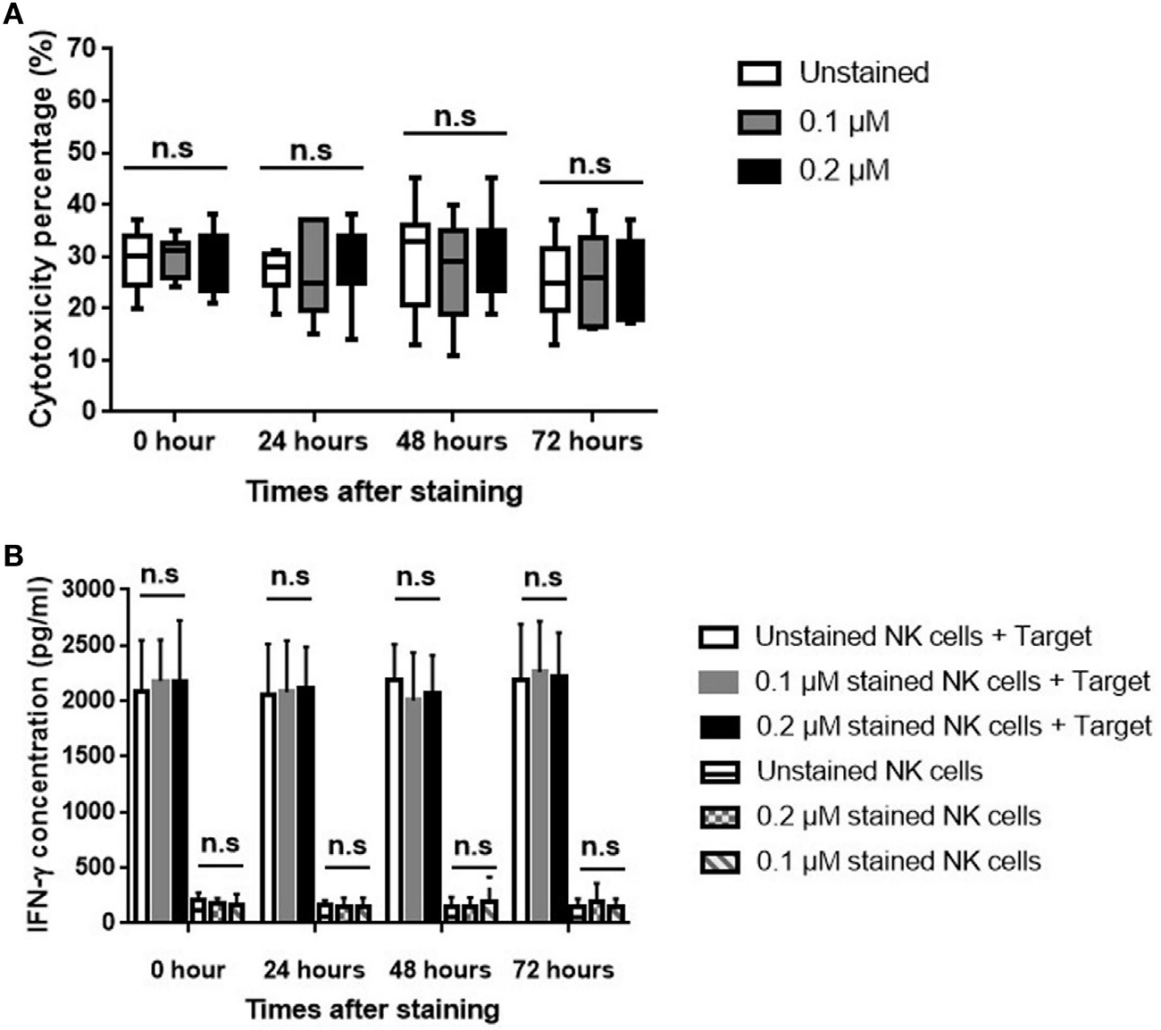
Human breast cancer MDA-MB-231 cell cytotoxicity of expanded ESNF13-stained natural killer (NK) cells. Expanded NK cells were stained with different concentrations of ESNF13 dye (0.1 and 0.2 µM) and the 4-h cytotoxicity and interferon-γ (IFN-γ) release against human breast cancer cell line MDA-MB-231 was assessed at each time point after staining with ESNF13 (0, 24, 48, and 72 h). **(A)** The 4-h cytotoxicity analysis was performed using a WST-8 proliferation assay. Stained NK cells killed cancer cells at a ratio of 0.5:1 (effector:target cells). **(B)** The concentration of IFN-γ (pg/ml) in the NK cell supernatant was detected using an enzyme-linked immunosorbent assay. The median, the first (Q1) and third (Q3) qualities, and the minimum and maximum are shown and n.s indicates non-significant (as determined by analysis of variance analysis).

The human breast cancer cell line MDA-MB-231 was used to evaluate the effect of ESNF13 on NK cell cytotoxicity at different time points (0, 24, 48, and 72 h). The cytotoxicity of NK cells was the same unstained cells and those stained with 0.1 and 0.2 µM ESNF13 (Figure 4A and Figure S3 in Supplementary Material). To investigate the effects of ESNF13 NIR fluorophores on NK cell activity, the IFN-γ release against MDA-MB-231 cells was measured. The IFN-γ release from NK cells stained with 0.1 and 0.2 µM of dye was with the same as the unstained samples at all time points (Figure 4B).

### Biodistribution of *Ex Vivo*-Expanded NK Cells on Non-Tumor-Bearing NSG Mice

**Figure 5:**
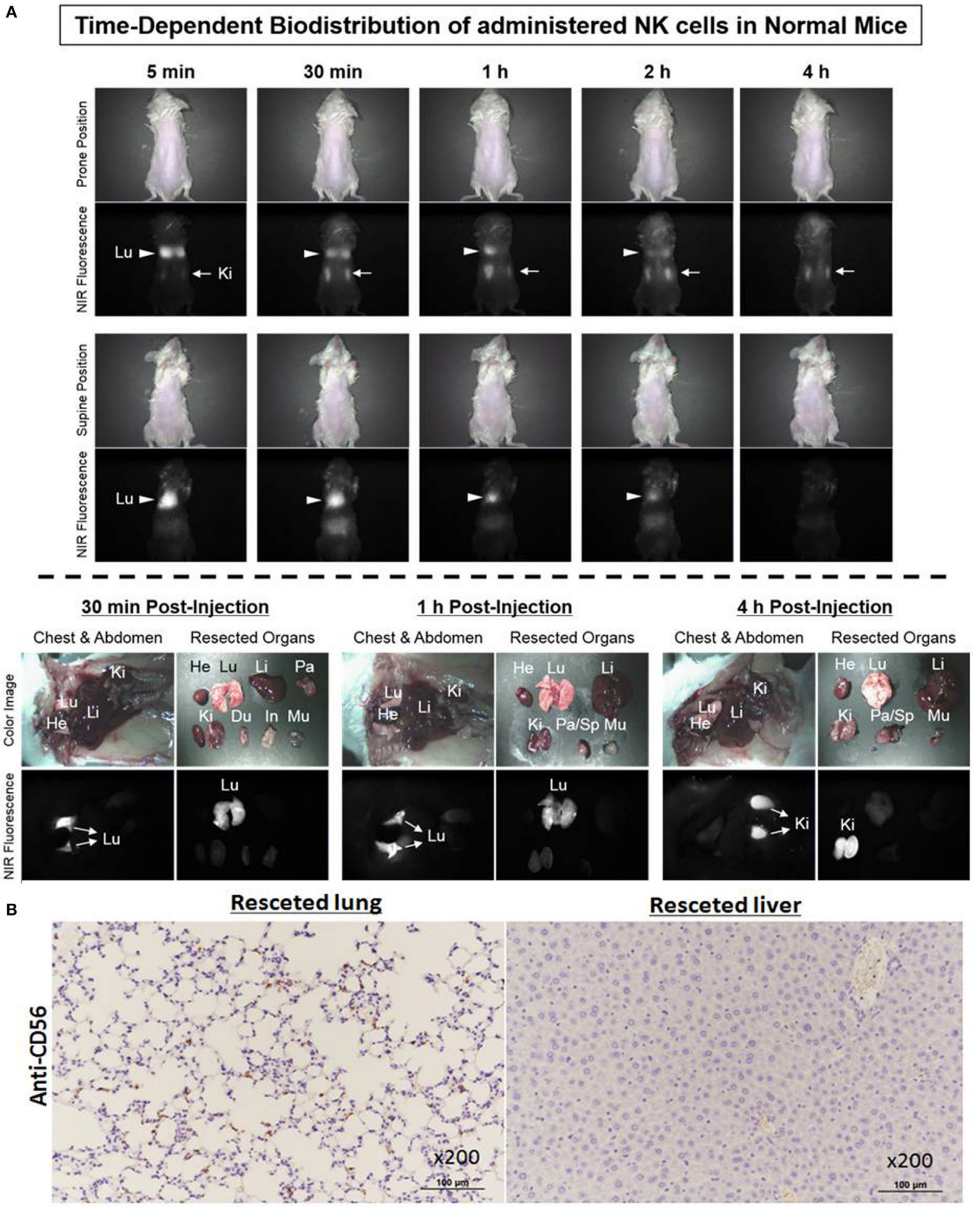
Biodistribution of *ex vivo*-expanded natural killer (NK) cells on non-tumor-bearing NSG mice. NK cells stained with 0.2 µM of ESNF13 dye were injected into normal NSG mice by intravenous routes. **(A)** The fluorescent signal was detected using a Mini-FLARE near-infrared (NIR) optical imaging system. After various time points, the mice were sacrificed and resected organs were explored. **(B)** The lung and liver were resected 4 h after injection of ESNF13-staind NK cells, where anti-CD56 was used to detect CD56^+^ NK cells. CD56^+^ NK cells were easily identified in the lung but did not populate in the liver.

To investigate the biodistribution of *ex vivo*-expanded NK cells using Mini-FLARE NIR optical imaging system, ESNF13-stained NK cells were intravenously injected into non-tumor-bearing NSG mice. Immediately after injection of NK cells labeled with ESNF13, infused NK cells mainly localized to the lungs, then the fluorescent signal increased in the kidney, and finally 4 h later, the signal markedly decreased in the lungs and fully accumulated in the kidney (Figure 5A). Mice were sacrificed at each post-injection time point (30 min, 1 h, and 4 h). At 30 min post-injection, the lungs had the brightest signal and the signal lasted until 1 h post-injection. Meanwhile, at 4 h post-injection, the signal disappeared after passing through the kidney. These results were consistent with the histopathological findings in the organ specimens (Figure 5B). NK cells labeled with CD56 antibody were easily identified in the lung of the NK cell-infused mice. By comparison, the lungs from the control mice without NK cell infusion did not have CD56^+^ NK cells (data not shown). In the liver resected from the NK cell-infused mice, CD56-labeled NK cells were not identified, consistent with the negative fluorescent signal observed using the NIR fluorescent systems.

### Real-Time Trafficking of NK Cells in Human Breast Cancer Xenograft Models

**Figure 6:**
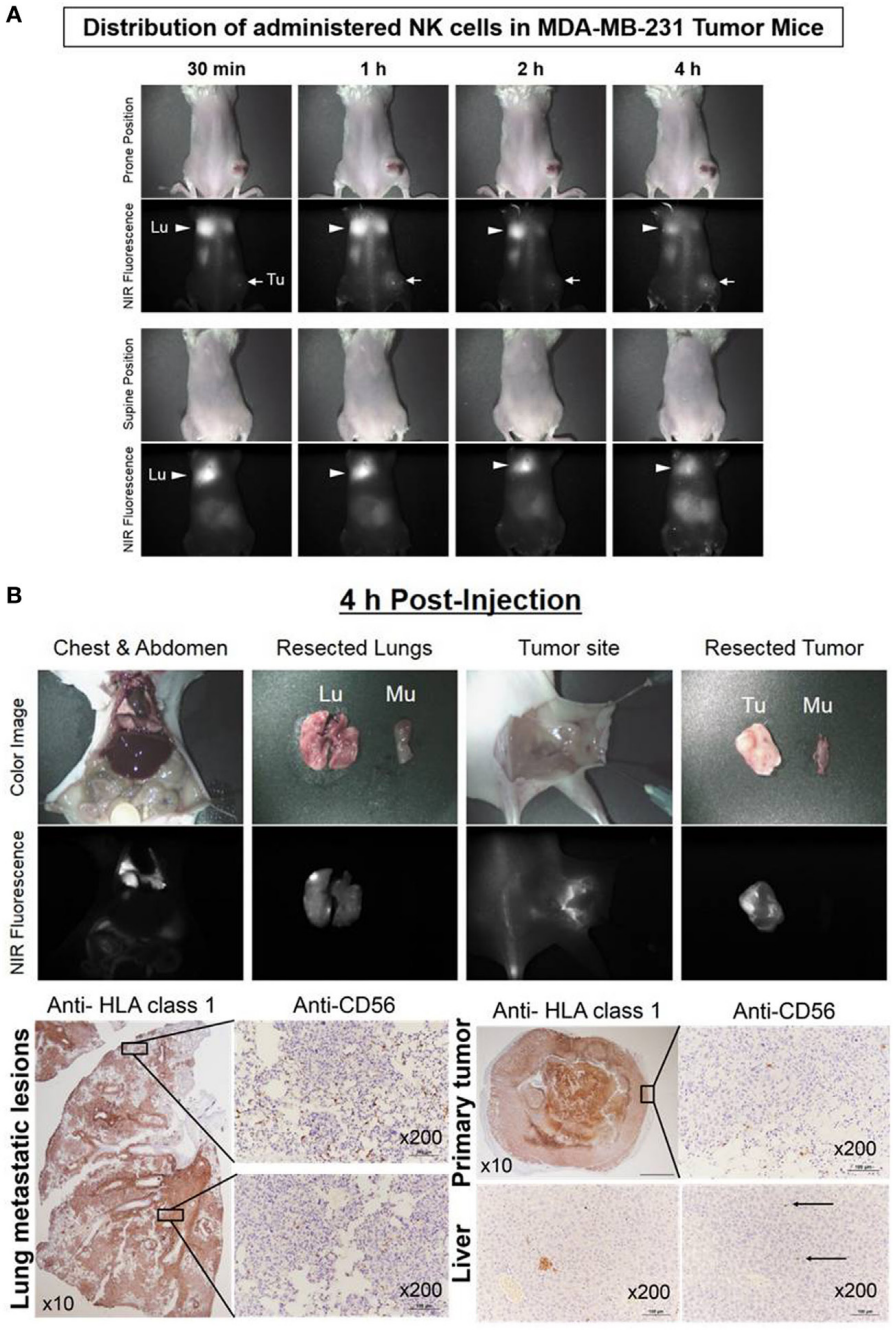
Real-time trafficking of natural killer (NK) cells in a human breast cancer xenograft model. Expanded NK cells were stained with 0.2 µM of ESNF13 and intravenously injected into MDA-MB-231 tumor-bearing NSG mice after 44 days tumor inoculation. **(A)** The fluorescent signal was detected using a near-infrared (NIR) fluorescent system. After 4 h, the mice were sacrificed and resected organs were explored. **(B)** The lung, liver, and tumor were resected 4 h after injection of ESNF13-stained NK cells and were stained with anti-HLA or anti-CD56 to detect the tumor and infiltrative CD56^+^ NK cells. The tumor cells were highlighted with HLA class 1 antibody and the localization of the NK cells corresponding to the scan imaging was demonstrated with CD56 immunohistochemistry. The lung showed a higher population of NK cells at the apex and the tumor specimen displayed an NK cell population mainly at the tumor margin. In accordance with fluorescence signal below detection limit, the liver had only micrometastatic tumors and few CD56^+^NK cells.

The human breast cancer MDA-MB-231 xenograft model was used to investigate the distribution and persistence of *ex vivo*-expanded NK cells. NK cells stained with ESNF13 were injected intravenously into MDA-MB-231 tumor-bearing NSG mice. The administered NK cells migrated to the lungs within 30 min. The fluorescence signal was dominant in both lungs and steady at 1 and 2 h. At 4 h post-injection, the signal was still dominant in the lung, with a slight increase in the tumors. Mice were sacrificed at 4 h after NK injection and the optical imaging signals were assessed in each organ (Figure 6A). Pathology examination confirmed that NK cells accumulated mainly in both lungs and substantially in the tumor sites (Figure 6B). Both lung had extensive and large metastatic tumor lesions. Consistent with the findings in the fluorescent scans, the apex area of the lung showed a higher population of NK cells compared with the lower lobe. Similarly, the resected tumor specimen with brighter signals at the rim compared with that in the middle region displayed an NK cell population that was mainly localized at the tumor margin. There were micrometastatic tumors and few CD56^+^ NK cells in resected liver specimen, in accord with fluorescence signal below detection limit.

### Real-Time Trafficking of NK Cell Migration to Primary Tumor Sites in a Human Breast Cancer Xenograft Model

**Figure 7:**
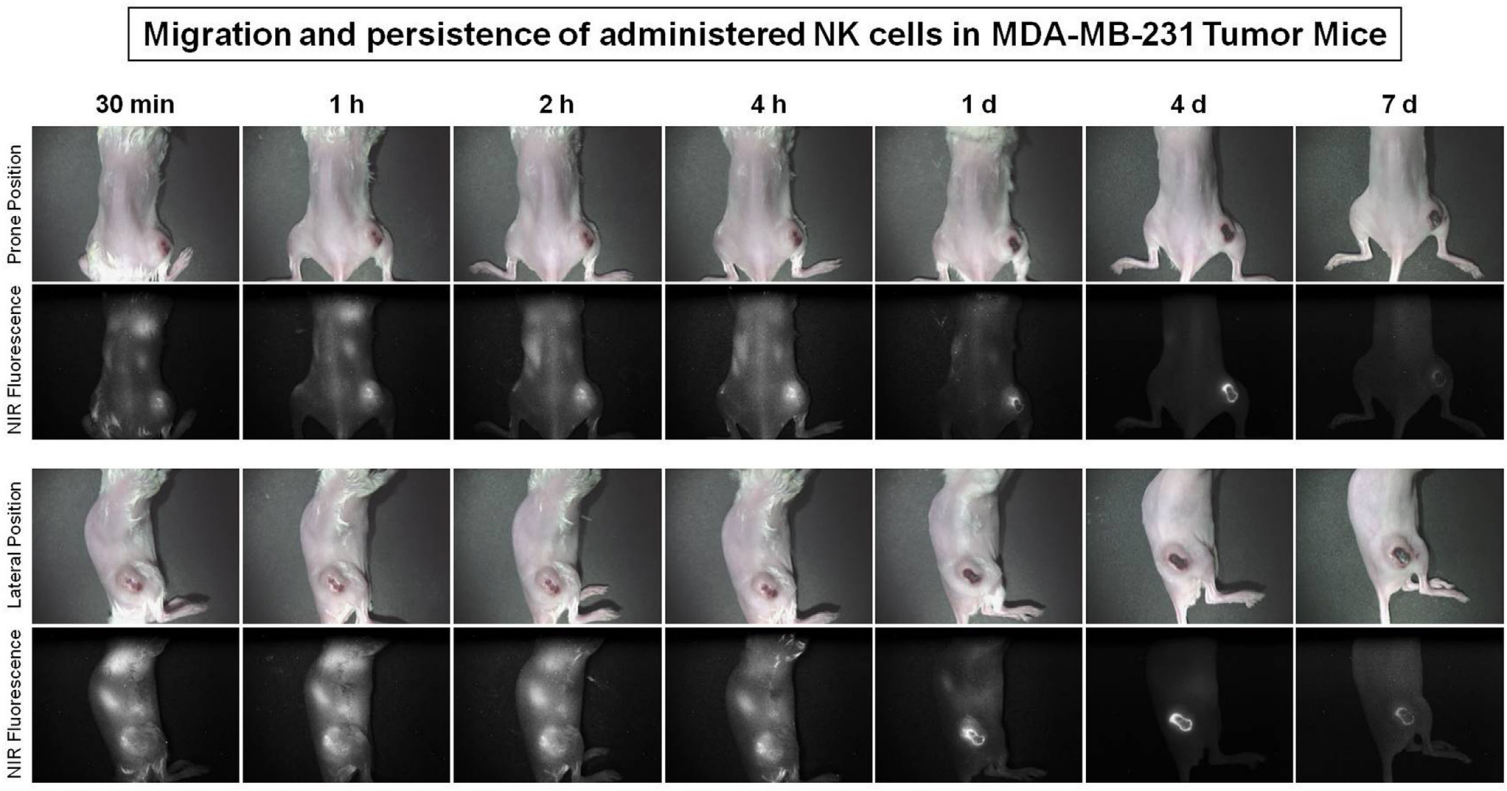
Migration of natural killer (NK) cells toward primary tumor sites in human breast cancer xenograft models. The MDA-MB-231 tumor-bearing mice (31 days after tumor injection) were used to investigate the trafficking of expanded human NK cells. Expanded NK cells were stained with ESNF13 dye and injected into MDA-MB-231 tumor-bearing NSG mice by intravenous. The fluorescent signal was visualized using a near-infrared (NIR) fluorescent system until day 7 post-injection.

To evaluate NK cell distribution according to tumor progression, stained NK cells were injected intravenously into the human breast cancer MDA-MB-231 tumor-bearing NSG mouse 31 days after tumor inoculation. Optical images were taken at different point of time for 7 days using a Mini-FLARE system. The administered NK cells migrated to the lung and tumor sites within 30 min and the signal dominated the tumor site after 1 h. The fluorescent signal of the tumors increased and remained steady at 1, 2, and 4 h, decreased on day 1 and day 4 scans, and then disappeared on day 7 scans (Figure 7).

## Discussion

Direct *in vivo* NK tracking can provide useful information about the distribution, persistence, and homing to tumor sites. We demonstrated that *ex vivo* NK cells circulated from the lung immediately after i.v. injection to the tumor site within 4 h post-injection in a TNBC xenograft mouse model. This is the first study to assess *ex vivo*-expanded NK cell accumulation at tumor sites using optical imaging. Limited research, in particular on solid tumor models, has assessed NK cell accumulation at tumor sites using other imaging modalities (7–9). A pioneering study regarding NK cell *in vivo* tracking using PET with radiotracer ^11^C reported that after 1 h injection, 4–30% of activated NK cells had accumulated in tumor sites in a xenograft fibrosarcoma mouse model (9). Genetically modified NK-92 cell line labeled with NIR dye showed increased fluorescence in tumors at 1.5 and 8 h post-injection and remained stable at 24 h in scans of the prostate cancer xenografts (7). In this study of human breast cancer xenograft models, *ex vivo*-activated and expanded NK cell migration to the tumor site increased at 30 min after i.v. injection, remained steady at 1, 2, and 4 h and decreased on day 1.

In this study, *ex vivo*-expanded NK cell tracking was successfully performed by labeling the NK cells with ESNF13 NIR fluorophores using simple co-culturing methods. Of several NIR fluorophores for optical fluorescence, the genetically modified NK cells labeled with fluorescent dye DiD (1,1-di-ocadecyl-3,3,3,3,-tetramethylindodicabocyanine) were successfully tracked in a preclinical study (7). However, no imaging agents or reporter genes that encode fluorescent proteins were evaluated for optical imaging of *ex vivo-*expanded NK cells. The ESNF13 NIR fluorophore used in this study is a non-targeted lipophilic probe that is adherent and diffuses to the phospholipid cell membrane bilayer (11). At a low dose range, ESNF13 NIR fluorophores had no statistically significant adverse effect on proliferation and cytotoxicity of *ex vivo*-activated NK cells in our results. Recently, there has been increasing interest in the improvement of NK cell-based cellular immunotherapy, such as the development of various protocols for *ex vivo* NK cell expansion, genetically engineered NK cells using a chimeric antigen receptor for tumor recognition and killing, and combinations with various cytokines for *in vivo* survival and proliferation. Our imaging approach can be useful for the development of new therapeutic NK cell-based immunotherapies using the rapid optical imaging of NK cell biodistribution and kinetics.

Unlike previous studies, we evaluated the migration pattern of non-targeted, *ex vivo*-activated, expanded NK cells without genetically engineered modification for the targeting of specific tumor antigens by optical imaging. Previous research showed that only 3–4% of non-activated lymphocytes accumulated to tumor sites after systemic administration of NK cells (9). The activated and expanded NK cells investigated in our study are highly cytotoxic to a variety of tumor types, with no genetic modification required to target specific tumor antigens (18). Thus, although these expanded NK cells have no motif specific to a tumor antigen, *ex vivo*-activated and expanded NK cells can substantially migrate toward tumors in comparison with inactivated lymphocytes. In the era of the immunotherapy revolution, NK cell-based immunotherapy has been evaluated in limited settings for hematologic cancer, minimal residual disease, or an adjuvant modality. In contrast to T cells, NK cells can directly bind to the tumor cell and induce cytotoxicity without graft-versus-host disease (3, 19). Highly cytotoxic, activated NK cell accumulation in solid tumors in our study is promising for efficient adoptive NK cell therapies against solid tumors resistant to other modalities or for combined therapy with chemotherapy, radiation, or immune check-point inhibitors. In future research, we aim to assess correlations between tumor control, optical imaging results, and therapeutic efficacy.

In our preclinical study, the biodistribution of NK cells labeled with NIR fluorophore, ESNF13, in the non-tumor-bearing NSG mouse had similar pattern as observed in clinical settings. Immediately after injection of NK cells labeled with ESNF13, the NK cells mainly localized in the lungs, then the fluorescence signal increased in the kidney, and finally, 4 h later, there was a marked decrease in signal in the lungs and full accumulation in the kidneys. The biodistribution and kinetics of allogenic NK cells have been evaluated in patients with renal cell carcinoma with metastasis (10, 20). Authors reported that ^111^In-labeled NK cells accumulated primarily in the lungs, then distributed to the liver, spleen, and bone marrow within 24 h, with only 4–8% of the whole-body activity in the lungs. At that time, it was observed that large metastatic lesions showed uptake of ^111^In-labeled NK and NK cells persistent in the blood up to 3 days, as determined by PCR (10). Unlike in the clinical setting, our labeled NK cells showed little liver accumulation. Although there are some differences in NK cells liver accumulation between the preclinical and clinical settings, our NSG mouse model, injected into human breast cancer MDA-MB-231 cells resulted in a metastatic pattern similar to that observed in human breast cancer patients (21). HLA class 1 was highly expressed on MDA-MB-231 cancer cells (22, 23) and as a potential marker for detection of human TNBC (24, 25). In this study, anti-human HLA-1 was used to confirmation the present of human MDA-MB-231 cancer cells in both primary tumor and metastasis sites. Using optical imaging, the *in vivo* distribution of exogenous NK cells in a lung metastatic TNBC model was easily achieved. Toward offering surgeons a real-time method of visualization, the imaging community has pursued various avenues of intra-operative imaging by translating current spectral imaging modalities from pre-operative setting, including computed tomography or PET. Although optical imaging system has a critical limitation in terms of penetration depth for whole-body imaging, intra-operative image-guided detection of various human tissues or diseases targeted by NIR fluorophores could help surgeon for accurate resection and preservation during surgery.

Interestingly, there are different patterns of NK cell distribution and homing to tumor sites between late and early phase metastatic breast tumor models. In this study of metastatic breast tumor-bearing mouse 44 days after tumor inoculation, optical imaging revealed an initial NK cell accumulation that was mainly observed in lung metastatic lesions and was steady at 1, 2, and 4 h post-injection. However, in early phase metastatic breast tumor model (31 days after tumor injection), the fluorescence signal of the tumors appeared at 30 min after NK cell injection, increased and remained steady until 4 h, decreased on both day 1 and day 4 scans, and then disappeared on day 7. The relationship between T-cell and NK cells have already reported in which NK cells can regulate the immunity response of T cells through cytokines and costimulatory molecules (26, 27), while T regulatory cells can also suppress NK cells activity (28). However, based on limitation of NSG mice model, which lacks T cells, B cells and NK cells, the effect of T cells on *ex vivo-*expanded NK cells migration patterns under physiological tumor microenvironment remain still unknown. In this study, we examined TNBC using MDA-MB-231 cells that lack the expression of estrogen, progesterone and HER2 receptors. There is an ongoing phase III study of a combination of anti-PD-1 with chemotherapy for patients with advanced TNBC (NCT03125902) and with early stage TNBC (NCT03197935). To date, a new therapeutic T-cell approach against solid tumors is more effective than using NK cells. However, the T-cell approach using check-point inhibitors, such as PD-1 or PD-L1, respond to only 18–28% of solid tumors (14, 29), and many studies have revealed that check-point inhibitors only have limitation to overcome immune escape at tumor microenvironments (30). Various *ex vivo* expansion protocols have been developed to generate large numbers of highly cytotoxic NK cells (18, 31, 32), but tumor control and treatment outcomes of activated and expanded NK cell-based therapy varies depending on the experimental design (1), indicating the determining the optimal dose and timing of NK cells, in combination with other modalities, may be of particular therapeutic value. Our optical imaging modality is easily applicable (i.e., it involves simple incubation with NIR fluorophores) and may provide useful information about tumor control and survival through serially rapid image acquisition for investigating the biokinetics of NK cells.

## Ethics Statement

The use of animals for this study was approved by the Institutional Animal Care and Use Committee of Chonnam National University (CNU IACUC-H-2017-52).

## Author Contributions

TU, KK, JM, HH, and MY participated in the living imaging experiments: KL, SA, and MY performed the pathologic data collection and analysis; MY and HH designed the study and analyzed imaging data; JM provide optical imaging system.

## Conflict of Interest Statement

All authors report no conflict of interest. The authors have no commercial, proprietary, or financial interest in the products or companies described in this article.

## Supplementary Material

Figure S1 Effect of ESNF13 on natural killer (NK) cell purity at different concentrations. The representative dot plot examples of stained or unstained CD56^+^CD3^−^ NK cells at different post-injection time points (0, 24, 48, and 72 h).

Figure S2 Alive and dead population of natural killer (NK) cells stained with near-infrared (NIR) fluorophores. **(A)** The representative density plot of PI staining of living and dead populations. **(B)** The representative density plot examples of alive and dead population of NK cells stained with 0.1 and 0.2 μM NIR fluorophores at different time intervals (immediately at 24, 48, and 72 h after staining).

Figure S3 Cytotoxicity at different E:T ratio of expanded natural killer (NK) cells stained with near-infrared fluorophores ESNF13 against MDA-MB-231 human breast cancer cell lines. Expanded NK cells were stained with different concentration of ESNF13 dye (0.1 and 0.2 μM) and checked the cytotoxicity activity and the IFN-γ release against breast human cancer cell lines MDA-MB-231 at different E:T ratio (0.5:1 and 1:1).
