# Supplementary figures and images for: Real-Time Tracking of Ex Vivo-Expanded Natural Killer Cells Toward Human Triple-Negative Breast Cancers
Source: Front Immunol. 2018 May 2;9:825. doi: 10.3389/fimmu.2018.00825 (PMC5941970; doi:10.3389/fimmu.2018.00825)

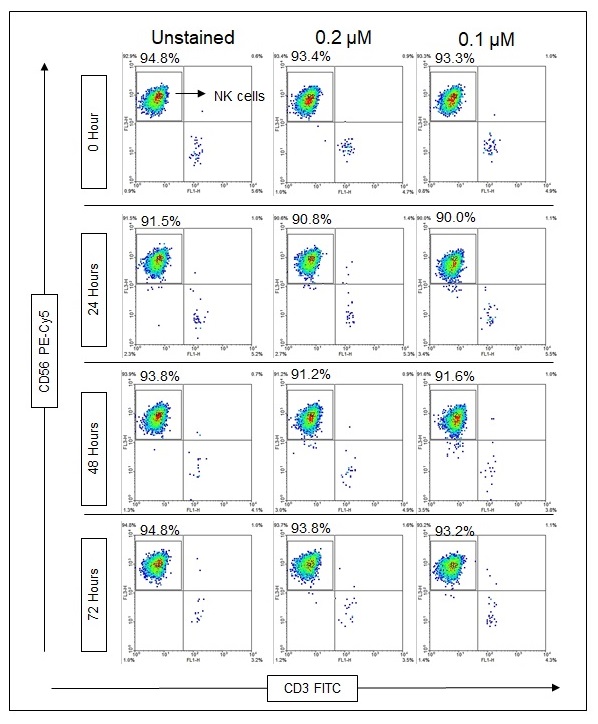

Supplement: Figure S1 — Effect of ESNF13 on natural killer (NK) cell purity at different concentrations. The representative dot plot examples of stained or unstained CD56+CD3− NK cells at different post-injection time points (0, 24, 48, and 72 h). [file Image_1.jpg]

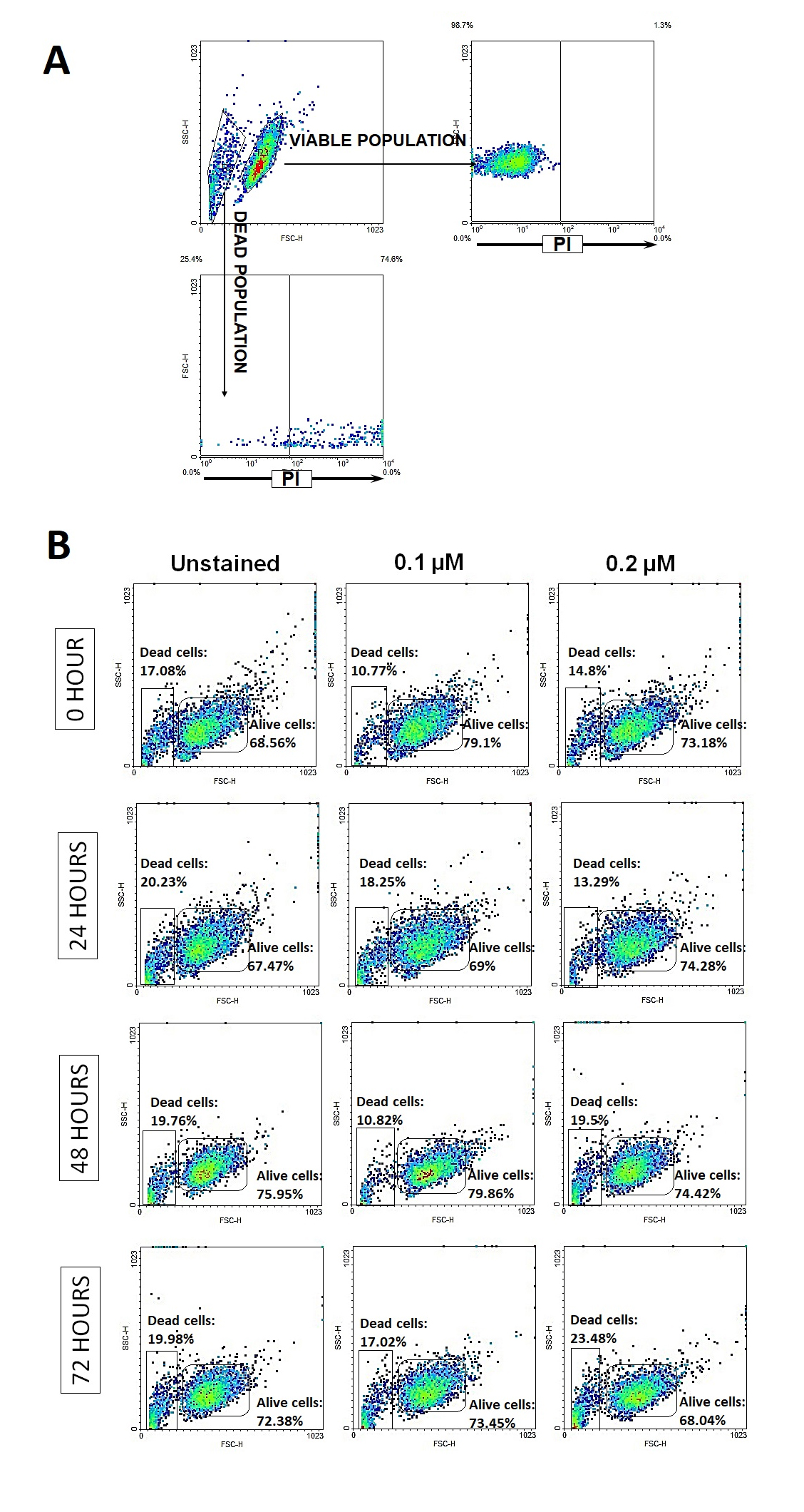

Supplement: Figure S2 — Alive and dead population of natural killer (NK) cells stained with near-infrared (NIR) fluorophores. (A) The representative density plot of PI staining of living and dead populations. (B) The representative density plot examples of alive and dead population of NK cells stained with 0.1 and 0.2 μM NIR fluorophores at different time intervals (immediately at 24, 48, and 72 h after staining). [file Image_2.jpg]

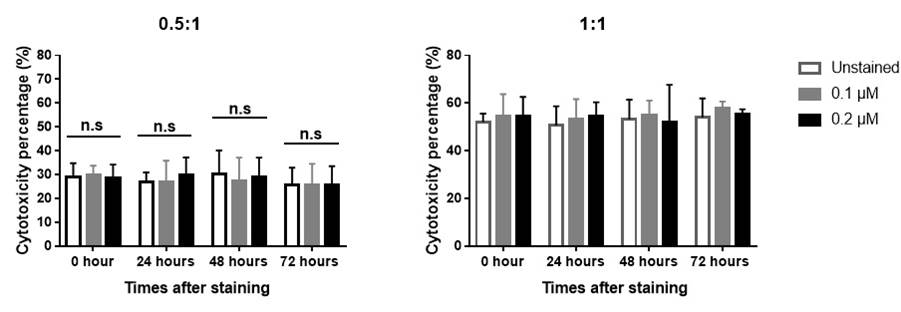

Supplement: Figure S3 — Cytotoxicity at different E:T ratio of expanded natural killer (NK) cells stained with near-infrared fluorophores ESNF13 against MDA-MB-231 human breast cancer cell lines. Expanded NK cells were stained with different concentration of ESNF13 dye (0.1 and 0.2 μM) and checked the cytotoxicity activity and the IFN-γ release against breast human cancer cell lines MDA-MB-231 at different E:T ratio (0.5:1 and 1:1). [file Image_3.jpeg]
